# Frailty prevalence and related factors in older adults from southern Brazil: A cross-sectional observational study

**DOI:** 10.6061/clinics/2020/e1694

**Published:** 2020-07-27

**Authors:** Jarbas Melo Filho, Natália Boneti Moreira, Audrin Said Vojciechowski, Simone Biesek, Paulo Cesar Barauce Bento, Anna Raquel Silveira Gomes

**Affiliations:** I Instituto Federal do Parana, Curitiba, PR, Brazil; II Departamento de Prevencao e Reabilitacao em Fisioterapia, Universidade Federal do Parana, Curitiba, PR, BR; III Programa de Mestrado e Doutorado em Educacao Fisica, Universidade Federal do Parana, Curitiba, PR, BR

**Keywords:** Frail Elderly, Prevalence, Evaluation

## Abstract

**OBJECTIVES:**

To determine the prevalence of frailty and the association of sociodemographic characteristics, clinical aspects, and functional capacity with the frailty status of community-dwelling older adults from Curitiba, Southern Brazil.

**METHODS:**

This cross-sectional observational study included 1,716 participants aged ≥60 years. Frailty was assessed using the Fried phenotype indicators of weakness, exhaustion, low activity, slowness, and weight loss. Sociodemographic characteristics, clinical aspects, and functional capacity and functionality were evaluated and compared between the sexes and the different frailty statuses (non-frail, prefrail, and frail). Multinomial logistic regression models were used to identify associations (*p*<0.05).

**RESULTS:**

A high prevalence of frailty (15.8%) and prefrailty (65.3%) were observed, and both were higher in female than in male individuals. The most predominant frailty criterion was weakness, followed by exhaustion. Compared with the non-frail elderly, the prefrail and frail elderly were older in age and presented more health problems, greater dependency for basic and instrumental activities of daily living, and reduced lower extremity strength performance and functional mobility. The highest proportion of illiterate individuals, individuals with 1-4 years of education, widowed individuals, polypharmacy, and possible cognition problems and diseases were seen in the frail elderly group. Moreover, the risk of being prefrail and frail was higher in those who were older and had more health problems, higher body mass index, and reduced lower extremity strength performance. Greater calf circumference and independence in activities of daily living were protective factors for prefrailty and frailty. Furthermore, lower functional mobility increased the chances of being frail.

**CONCLUSIONS:**

The prevalence of frailty was more pronounced in female than in male individuals, mainly because of a decline in force. Prefrailty was 4 times more prevalent than frailty, and the presence of health problems and reduced functional capacity increased the chances of being prefrail and frail.

## INTRODUCTION

Physical frailty is a biologic syndrome characterized by a progressive loss of strength, endurance, and physiological responses, which increases an individual’s vulnerability, dependency, and/or mortality when exposed to a stressor (1). In addition, cognition problems and chronic diseases may be present (2). These aspects compromise the functional capacity of older adults and increase disability and the risk of falls (3). Early identification of factors associated with frailty in older adults can guide effective prevention and treatment strategies (4).

The prevalence of frailty was shown to be 17.4%, whereas that of prefrailty was 49.3%, according to a recent worldwide review. Moreover, the review also showed that prevalence was higher in older people from the upper middle-income countries than in those from high-income countries (5). Frailty prevalence in Latin America and the Caribbean was twice as high (19.6% of frailty) as that in developed countries, probably because frailty is associated with lower income and education, worse health status, and higher rates of disability and chronic diseases (6-8).

Epidemiological studies have reported that the prevalence of frailty and prefrailty ranged from 8.7-9.7% to 46.3-51.8%, respectively, in the different regions of Brazil (north, northeast, south, and southeast) (8-11). However, behavioral, sociocultural, and biological aspects related to regional discrepancies do not permit the extrapolation of frailty prevalence. For example, investigation results from a south Brazilian city showed different features from that obtained from Curitiba, the capital of Paraná, with respect to the proportion of elderly (10% *versus* 11.3%), the human development index (HDI) (0.78 *versus* 0.82), and other sociodemographic aspects (12). Moreover, Curitiba is one of the cities with the highest longevity among the Brazilian metropolises. Furthermore, the aging index of the Paraná state in 2016 was higher than that of Brazil overall (44.2 *versus* 39.7 points) (13). Thus, as longevity was more pronounced in this population, it was necessary to investigate the frailty prevalence and its associated factors in the older people from Curitiba, in order to guide public health strategies.

Moreover, functionality, assessed mainly by lower limb strength, is essential to accomplish activities of daily living (ADL). This still needs to be investigated further, mainly with regard to its association with frailty, its contribution to guide the prescription of physical exercises, the principal intervention in the prevention and treatment of frailty (9,).

Thus, the aims of this cross-sectional study were to determine the prevalence of frailty and to assess whether the sociodemographic characteristics, clinical aspects, and functional capacity and functionality were independently associated with the frailty status of the older community-dwelling adults from Curitiba, Southern Brazil.

## MATERIALS AND METHODS

### Population and sample

This cross-sectional observational study was conducted in Curitiba, Paraná, Brazil, which is a city with 197,965 inhabitants aged ≥60 years (12). In 2016, the Municipal Health Secretary served 100,194 older adults across 9 city districts. The sample size for the present study was calculated using the Epiinfo calculator developed by the Centers for Disease Control and Prevention, with the following parameters: (i) the population was distributed among the 9 districts; (ii) 95% confidence level; (iii) sampling error of 3%; (iv) 50% of anticipated frequency, considering the maximum variance; and (v) design effect of 1.5 to correct the sample selection biases. Therefore, the initial sample size estimated was 1,593 older adults. All health units (HUs) in the 9 districts of the city were involved, and the number of participants from each HU was determined to obtain a proportional fraction of older adults assisted by the Municipal Health Secretary.

The Dom Bosco College (number 1,203,602) and the Municipal Health Secretary (number 1,254,580) Ethics Committees approved the procedures for this study, according to the Resolution of the National Health Council 466/2012. All individuals provided written informed consent to participate in the study.

Individuals aged <60 years and those suffering from neurological or musculoskeletal problems that limited their participation in all procedures (mainly, screening for physical frailty) were not included in this study. Thus, from a total of 1,794 older adults who agreed to participate in the study, 78 were excluded: 32 (1.78%) for not having completed all assessments (questionnaires and functional tests) and 46 (2.56%) for duplicate data. Finally, 1,716 older adults were included in the study, of whom 1299 (75.7%) were female and 417 (24.3%) were male.

### Instruments and procedures

Data were collected from March to September 2016 via face-to-face interviews conducted in the HU randomly selected from the 9 city districts. The number of groups per region was calculated so as to obtain a participant number proportional to the size of the city district. Older adults were invited personally from the HU to participate in this study. Finally, 16 HUs from 5 city districts were selected. All assessments were performed on a single day over an average duration of 1.5 hours. The sequence of the evaluations was standardized.

### Sociodemographic and clinical characteristics

Information regarding age, sex, education, and marital status was recorded during the interviews. Cognitive screening was assessed using the Mini Mental State Examination (17). The cutoff values with respect to education levels for Brazilian older adults were as follows: 20 points for illiteracy, 25 points for 1-4 years of education, 26 points for 5-8 years of education, 28 points for 9-11 years of education, and 29 points for >11 years of education (18). Data on clinical characteristics, such as the number of health problems (self-reported diseases), self-reported polyphamarcy (no=0-4 medications, yes=≥5 medications) (19), nutritional status by assessing body mass index (BMI) and calf circumference (CC), were obtained during the interview and from physical assessments.

### Functional capacity and functionality

To evaluate the independence in performing basic ADL and instrumental ADL (IADL), the Brazilian Portuguese versions of the Katz Index (20) and Lawton questionnaire (21) were used. Higher scores on the questionnaires indicated higher independence. The functional tests carried out included the five times sit-to-stand test, involving rising from an armless chair with both hands crossed against the chest (22), to assess lower extremity strength performance and the timed up and go test, wherein the person was instructed to rise from a chair, walk 3 m, turn around, and return to a sitting position at usual gait speed (23), to assess functional mobility. In these tests, lesser time taken for execution indicated better functionality.

### Physical frailty

The Fried phenotype was used to assess physical frailty and included the following indicators: unintentional weight loss (≥10 pounds or ≥5% of body weight in last year); self-reported exhaustion, which was investigated using two questions from the Center for Epidemiological Studies Depression Scale; low activity level (kilocalories expended per week, adjusted by sex), which was evaluated using the Minnesota Leisure Time Activities questionnaire (24); slowness (4 m gait test at usual speed adjusted for sex and height); and weakness (grip strength that was adjusted for sex and BMI), which was calculated using a manual dynamometer. The thresholds for low activity, slowness, and weakness were calculated on the basis of the values from the current sample to estimate the cutoff points for this study. The cutoff points for the exhaustion and unintentional weight loss criteria were based on those described by Fried et al. (8) and were also based on the frailty classification (frail ≥3 criteria present, prefrail=1 or 2 criteria present, and non-frail=0 criteria present).

### Data analysis

Descriptive statistics were in the form of mean and standard deviation (continuous data) and absolute and relative frequencies (categorical data). The Kolmogorov-Smirnov test demonstrated that all variables were not normally distributed. The Mann-Whitney U-test, Kruskal-Wallis test (continuous data), and chi-squared test (categorical data) were performed to compare sociodemographic characteristics, clinical aspects, and functional capacity and functionality between the sexes (male and female) and the different frailty statuses (non-frail, prefrail, and frail).

Multinomial logistic regression was used to estimate the outcome variables (sociodemographic characteristics, clinical aspects, and functional capacity and functionality) to predict the frailty status (non-frail, prefrail, and frail). Odds ratio values and 95% confidence intervals were calculated. All statistical procedures were performed using Statistical Package for the Social Sciences (version 22), and the significance level was set at *p*<0.05.

## RESULTS

The cutoff points for the physical frailty criteria of low activity, slowness, and weakness, on the basis of the current sample set, are presented in Table 1.

The study sample consisted of 1,716 older adults aged between 60 and 96 years, with a mean age of 71.0 (7.3) years, and was made up of 1,299 females and 417 males (mean age 70.8 [7.3] years and 71.5 [7.3] years, respectively). The prevalence of the different frailty statuses was as follows: 18.9% non-frail, 65.3% prefrail, and 15.8% frail. There was a higher percentage of non-frail males (40.0%) than females, but the percentage of prefrail (69.3%) and frail females (18.6%) was more than that of males. The most predominant frailty criterion was weakness, followed by exhaustion, slowness, low activity, and weight loss in the total sample, as well as in females. However, in males, the most prevalent frailty criteria were weakness, low activity, slowness, exhaustion, and weight loss.

Regarding sociodemographic characteristics, clinical aspects, and functional capacity and functionality in female individuals, higher scores for the presence health problems and BMI were observed. Male individuals showed more independence in ADL, whereas female individuals showed more independence in IADL. Additionally, the male individuals had greater handgrip strength than did female individuals.

The most prevalent indicators for frailty in females were widowed status, 1-4 years of education, polypharmacy, prefrail and frail statuses, and weakness and exhaustion. On the other hand, the most prevalent indicators in males were marital status, >8 years of education, no polypharmacy, and non-frail status. A detailed description of sociodemographic characteristics, clinical aspects, and functional capacity and functionality stratified by sex is shown in Table 2.

As can be seen from Table 3, advanced age, greater number of health problems, lowest scores on the Mini Mental State Examination, highest BMI, greatest dependence in ADL and IADL, and worst functionality (lower extremity strength performance and functional mobility) were more predominantly seen in older people classified as prefrail and frail.

As regards educational level, marital status, number of medications, and health problems, it was observed that the non-frail elderly were educated to the highest level (>8 years), were married, and did not have polypharmacy. Prefrail older adults were characterized by 1-4 years of education, while the frail elderly were usually illiterate or had only 1-4 years of education and were also characterized by widowed status, polypharmacy, and more health problems (such as hypertension, diabetes, depression, dementia, and Parkinson; Table 3).

Logistic regression analysis revealed that sociodemographic characteristics, clinical aspects, and functional capacity and functionality could predict prefrailty and frailty in older people (Table 4).

Advanced age, the presence of more health problems and higher BMI, as well as reduced lower extremity strength performance were considered as risk factors for prefrailty. However, greater CC and independence in ADL were protective factors for prefrailty.

Moreover, the chance of being frail increases with advancing age, the presence of more health problems and higher BMI, and reduced lower extremity strength performance and functional mobility. The protective factors for frailty were higher CC and greater independence in ADL.

## DISCUSSION

In the present study, the prevalence of physical frailty was 15.8%, prefrailty 65.3%, and non-frailty 8.9%. A difference in the prevalence rates (8.7-9.1% frail and 46.3-49.6% prefrail) was noted in the older community-dwelling adults from the cities of southeastern Brazil, showing a disparity between the south and southeast regions of the country (9,11,25). Additionally, a previous study with 3,478 older adults from 7 Brazilian cities chosen for convenience showed different results (9.1% frail and 51.8% prefrail) than those in the present study (10). Regional disparities can be explained by the differences in the HDI, aging index, and access to health assistance among the elderly.

Although the northern region of Brazil presents a lower HDI when compared with the southern region, suggesting differences in the prevalence of physical frailty, a similar prevalence was found in older adults from Santa Cruz, Rio Grande do Norte, Brazil (17.1% frail and 60.1% prefrail) (16). Furthermore, it is suggested that frailty is a health problem associated with older age (8), and both studies under discussion demonstrated that advanced age, the presence of comorbidities, and dependence for ADL were risk factors for the development of frailty.

Moreover, it was identified that female individuals showed a higher prevalence of prefrailty and frailty than did male individuals, corroborating the results of other studies (6,10). Although, notably, females live longer than males, their health status could be comparatively poorer due to higher environmental influences on frailty or due to the effect of lifestyle, thus increasing their vulnerability to subcellular mechanisms leading to increased recovery time (26). Furthermore, the relationship between frailty and female sex has been described in the literature as females show greater propensity for developing sarcopenia when compared with males (8). The identification of sex-associated risks of frailty is useful to address biological and lifestyle factors that contribute to the physical vulnerability for frailty prevention.

Weakness was the most common manifestation of frailty criteria reported in studies that evaluated physical frailty in the elderly (10,11,25,27). It is important and recommended to maintain and/or increase muscle strength to prevent and/or reverse frailty (1).

In the current study, exhaustion was the second most prevalent criterion, in line with the results from Sousa-Santos et al. (27), who investigated physical frailty in 1457 older people from Portugal. Similar to the results of that study, we also found a higher prevalence of overweight individuals among the prefrail and frail elderly, which could explain the relationship between the low level of physical activity, which is in turn associated with being overweight, and higher reports of tiredness. Despite malnutrition and the risk of malnutrition often being present in elderly people with physical frailty (28), there is strong evidence that excessive body adiposity contributes to the development of frailty by reducing the capacity of individuals to perform physical activities and increasing metabolic instability (29).

Our findings corroborate with the studies that investigated older Brazilian adults and reported higher percentages of frailty and prefrailty in individuals with advanced age, lower levels of schooling, and widowed status (9,11,25). Moreover, a review highlighted that the main sociodemographic factors positively associated with frailty were age, female sex, and black race, while schooling and income showed inverse associations, that is, the lower the schooling level and income were, the worse was the status of frailty in older adults (14). Therefore, greater attention should be given to females >70 years with lower levels of schooling and widowed status, and this can be done by screening for frailty, conducting orientation programs, and providing interventions (1,4).

Overall, our study results demonstrated that females and frail older adults presented more health problems, had higher BMI, and showed polypharmacy. However, Calado et al. (25) evaluated independent elderly people in Ribeirão Preto, São Paulo, Brazil, and did not find any significant relationship between the frailty status and number of medications used. However, despite the regional differences observed in these studies, reducing polypharmacy to prevent and reverse physical frailty has been strongly recommended (1). Polypharmacy may lead to adverse effects and drug interactions, contributing to geriatric syndromes, disability, and increasing mortality (30).

The worst performance in the cognitive screening test in our study was found among prefrail and frail older adults. Other studies have observed that worse cognitive status are associated with physical frailty (9,10,14,31). Furthermore, Macuco et al. (31) reported that cognitive status could be influenced by age, education, family income, and frailty status. Thus, neuropsychological tests should be used for thoroughly investigating the cognitive status in frail or prefrail older people, especially when they present lower scores in cognitive screening. Moreover, the combination of lower cognitive status and polypharmacy could lead to negative clinical outcomes (30).

Diseases such as hypertension, diabetes, depression, dementia, and Parkinson were more prevalent in those with frail status, while osteoarthritis was more prevalent in those with prefrail status. Mello et al. (14) reviewed the socioeconomic and health factors associated with frailty and reported that the main clinical factors associated with frailty were cardiovascular diseases, number of comorbidities, depressive symptoms, high BMI, smoking, and use of alcohol.

Calado et al. (25) found associations between frailty and stroke, diabetes, neoplasia, osteoporosis, and urinary and fecal incontinence. They further added that frail older adults recorded more medical visits and had a greater chance of hospitalization in 1 year. Sousa et al. (16) investigated older adults from the northeastern region of Brazil and showed that frailty was associated with the presence of comorbidities, osteoporosis, vascular accidents, depression, and falls. Poor self-rated health was an additional factor associated with frailty, as evidenced by many studies (9,11,14,16).

In the present study, high BMI was a predictive factor of prefrailty and frailty. This contrast can be explained by the fact that excessive adiposity can lead to decreased physical functions, such as reduced muscle strength, and contribute to physical frailty (29).

Reduced strength/power in the lower limbs was another predictive factor of prefrailty and frailty. This result suggests that decline in muscle strength would be a common factor in frailty, but only functional mobility was a predictive factor in frail older people. This can be explained by the complexity associated with the movements of standing up, walking, turning back, and sitting down (32,33).

Our data showed that a greater CC was considered as a protective factor for prefrailty and frailty. In the current study, CC has been demonstrated to have a positive relationship with muscularity; hence, we hypothesized that the elderly with greater CC had greater muscle mass and strength, and consequently more functional independence. Landi et al. (34) also found that higher CC was associated with lower prevalence of frailty and greater functional independence in community-dwelling older people. However, CC should be analyzed carefully in cases such as ours where the sample of presents with a high BMI. Thus, other methods such as ultrasound and magnetic resonance imaging might be recommended to measure the muscle mass.

The relationship among functional independence, disability, and frailty was also reported in other studies, wherein higher frailty status, older age, and the presence of many comorbidities were associated with a higher level of dependence (9,). Vieira et al. (11) evaluated older adults from Belo Horizonte, Minas Gerais, Brazil, and observed that the frail elderly showed greater IADL limitations. In addition, the relationship between frailty and the use of gait devices, occurrence of falls, and hospitalization was also reported.

Furthermore, functionality, which is directly involved with functional capacity, was associated with frailty in the present study. Lustosa et al. (35) showed that prefrail and frail elderly had the worst functional performance in the “Timed up and go” test and IADL, reinforcing that frailty compromises the functionality of the elderly. The functional changes detected in frail older adults might be reversible upon early detection, which can then help guide the prevention and treatment strategy, along with recommendation for physical exercise and protein supplementation (1,4,36,37).

The findings of the present research recommend functional tests for the assessment of older people, especially their lower limb strength, because reduced lower extremity strength performance increases the chances of prefrailty and frailty, while lower functional mobility increases the chances of frailty.

The limitation of this study as well as other prevalence studies is the cross-sectional design, because it is not possible to determine whether the exposure preceded the effect. Thus, it is recommended that future studies with longitudinal design be performed to estimate the incidence of physical frailty. Furthermore, the participants were investigated only at the HU, which may have limited the evaluation of older people with greater limitations and worse clinical and sociodemographic conditions, like those under domiciliary healthcare and those not assisted at the HU. Therefore, these aspects may have affected the prevalence of frailty and other findings obtained in this study. Age-related changes in cognition can lead to variations in self-reported assessments, which can be considered a bias in this study.

The present study presents important clinical applications and could be recommended to screen physical frailty in community older adults, as over half of the study sample presented as prefrail. Health professionals should be attentive towards clinical conditions, such as hypertension, diabetes, depression, dementia, and Parkinson, polypharmacy, and low functional capacity (strength performance, functional mobility, and dependence in ADL and IADL) due to the association with physical frailty syndrome. There must also be emphasized interventions to improve muscle strength, as it was the most common frailty criterion found in the present study. Thus, physical exercises should be performed to avoid adverse health conditions. The outcomes can contribute to guide public health policies related to older adults.

## CONCLUSION

The prevalence of frailty and prefrailty was 15.8% and 65.3%, respectively, and was higher in females than in males. The most predominant frailty criterion was weakness, followed by exhaustion. Prefrail and frail elderly were older, presented more health problems, had greater dependency for ADL and IADL, demonstrated lesser inferior extremity strength performance, and had lower functional mobility than did non-frail older adults. The frail status was most prevalent among older illiterates, those with 1-4 years of education, widowed status, polypharmacy, and with diseases such as hypertension, diabetes, dementia, depression, and Parkinson. Chances of being prefrail and frail increases with advancing age, more health problems, higher BMI, and lesser inferior extremity strength performance. Greater CC and independence in ADL are protective factors for prefrailty and frailty. Lower functional mobility raises the chances of frailty.

### Bullet Points

- The prevalence of frailty was high in older adults from southern Brazil.
- Prefrailty was four times more prevalent than frailty.
- The prevalence of frailty and prefrailty was higher in females than in males.
- Most frequent frailty criteria were weakness and exhaustion.
- The chance of being prefrail and frail increases with lesser functional capacity.

## AUTHOR CONTRIBUTIONS

Melo Filho J, Moreira NB, Vojciechowski AS, Biesek S, Bento PCB and Gomes ARS contributed to the study conception and design, data acquisition, analysis and interpretation, manuscript drafting and critically review for important intellectual content. All of the authors approved the manuscript final version.

## Figures and Tables

**Table 1:** Cutoff values for low activity, weakness, and slowness in older adults from Curitiba, Parana, Brazil (n=1,716).

Frailty criteria	Cutoffs	
a **Low activity** (Minnesota, kcals/week) Stratified by sex	Males: <206	
	Females: <440	
a **Weakness** (HGS, kgf) Stratified by sex and BMI quartiles (kg/m^2^)	Males	Females
	BMI≤24.5; HGS≤21.0	BMI≤24.5; HGS≤21.3
	BMI>24.5≤27; HGS≤25	BMI>24.5≤27.5; HGS≤25.1
	BMI>27.5≤30; HGS≤27.7	BMI>27.5≤31.4; HGS≤28.1
	BMI>30; HGS≤30.9	BMI>31.4; HGS≤32.5
b **Slowness** (GS, seconds)Stratified by sex and medium height (cm)	Males	Females
	Heights ≤168; GS≥4.5	Heights ≤155 cm; GS≥4.6
	Heights >168; GS≥4.2	Heights >155 cm. GS≥4.2

a The values are 20^th^ percentile of the total sample.

b The values are 80^th^ percentile of the total sample.

BMI, body mass index; HGS, handgrip strength; GS, gait speed; kgf, kilogram force.

**Table 2:** Sociodemographic characteristics, clinical aspects, functional capacity and functionality, and frailty criteria and status stratified by sex in older adults from Curitiba, Parana, Brazil (n=1,716).

Continuous variables	Total(n=1,716)	Females(n=1,299)	Males(n=417)
	Mean (SD)	Mean (SD)	Mean (SD)
**Sociodemographic and clinical aspects**			
Age (years)	71.0 (7.3)	70.8 (7.3)	71.5 (7.3)
Health problems (n)	2.3 (1.4)	2.5 (1.4)**	1.7 (1.3)
Cognition screening (score)	24.4 (4.5)	24.5 (4.3)	24.0 (5.0)
BMI (kg/m^2^)	28.1 (5.1)	28.3 (5.3)*	27.5 (4.3)
CC (cm)	35.9 (4.1)	35.9 (4.2)	36.1 (3.8)
**Functional capacity and functionality**			
ADL (score)	5.6 (0.8)	5.5 (0.8)	5.7 (0.7)**
IADL (score)	18.7 (3.2)	19.0 (2.8)**	17.8 (3.9)
Handgrip strength (kgf)	24.6 (8.3)	21.8 (5.5)	33.4 (9.3)**
Lower extremity strength performance (seconds)	12.4 (4.9)	12.4 (4.9)	12.3 (4.8)
Functional mobility (seconds)	10.1 (4.5)	10.1 (4.6)	10.1 (4.5)
Gait speed (seconds)	3.9 (2.0)	3.9 (2.1)	3.8 (1.7)

Categorical variables	Total	Females	Males
	n (%)	n (%)	n (%)
**Education level**			
Illiterate	145 (8.4)	108 (8.3)	37 (8.9)
1 to 4 years	702 (40.9)	555 (42.7)*	147 (35.3)
5 to 8 years	288 (16.8)	216 (16.6)	72 (17.3)
>8 years	581 (33.9)	420 (32.3)	161 (38.6)*
**Marital status**			
Single	161 (9.4)	125 (9.6)	36 (8.6)
Married	735 (42.8)	468 (36.0)	267 (64.0)**
Widowed	596 (34.7)	547 (42.1)**	49 (11.8)
Separated and Divorced	224 (13,1)	159 (12,2)	65 (15,6)
**Polypharmacy**			
No	1059 (61.7)	771 (59.4)	288 (69.1)**
Yes	657 (38.3)	528 (40.6)**	129 (30.9)
**Frailty States**			
Non-frail	325 (18.9)	158 (12.2)	167 (40.0)**
Prefrail	1120 (65.3)	900 (69.3)**	220 (52.8)
Frail	271 (15.8)	241 (18.6)**	30 (7.2)
**Frailty criteria**			
Exhaustion	351 (20.5)	282 (21.7)*	69 (16.5)
Low activity	342 (19.9)	259 (19.9)	83 (19.9)
Weight loss	245 (14.3)	194 (14.9)	51 (12.2)
Weakness	1128 (65.7)	1036 (70.8)**	92 (22.1)
Slowness	347 (20.2)	267 (20.6)	80 (19.2)

SD, standard deviation; CC, calf circumference; BMI, body mass index; ADL, activities of daily living; IADL, instrumental activities of daily living; kgf, kilogram force;

* *p*<0.05;

** *p*<0.01.

**Table 3:** Comparison between frailty status and sociodemographic characteristics, clinical aspects, and functional capacity and functionality in older adults from Curitiba, Parana, Brazil (n=1,716).

Continuous variables	Non-frail(n=731)	Prefrail(n=875)	Frail(n=110)
	Mean (SD)	Mean (SD)	Mean (SD)
**Sociodemographic and clinical aspects**			
Age (years)	68.8 (6.4)^a^,^b^	71.0 (7.1)^c^	73.3 (8.3)
Health problems (n)	1.9 (1.3)^a^,^b^	2.4 (1.4)^c^	2.6 (1.5)
Cognition screening (score)	25.7 (4.0)^a^,^b^	24.6 (4.3)^c^	22.1 (5.2)
BMI (kg/m^2^)	26.4 (3.9)^a^,^b^	28.4 (5.0)	29.0 (6.2)
CC (cm)	35.9 (3.7)	36.1 (4.0)^c^	35.4 (4.9)
**Functional capacity and Functionality**			
ADL (score)	5.8 (0.7)^a^,^b^	5.6 (0.7)^c^	5.2 (1.1)
IADL (score)	19.6 (2.3)^a^,^b^	19.0 (2.8)^c^	16.5 (4.3)
Lower extremity strength performance (seconds)	10.4 (3.4)^a^,^b^	12.0 (4.1)^c^	16.3 (6.8)
Functional mobility (seconds)	8.4 (2.8)^a^,^b^	9.6 (3.3)^c^	14.0 (7.5)

Categorical variables	Non-frail	Prefrail	Frail
	n (%)	n (%)	n (%)
**Educational level**			
Illiterate	20.0 (6.2)	92.0 (8.2)	33.0 (12.2)*
1 to 4 years	98.0 (30.2)	479.0 (42.8)*	125.0 (46.1)*
5 to 8 years	56.0 (17.2)	191.0 (17.1)	41.0 (15.1)
>8 years	151 (46.5)*	358 (32.0)	72 (26.6.)
**Marital status**			
Single	29.0 (8.9)	108.0 (9.6)	24.0 (8.9)
Married	185.0 (56.9)*	467.0 (41.7)	83.0 (30.6)
Widowed	67.0 (20.6)	402.0 (35.9)	127.0 (46.9)*
Separated and Divorced	44.0 (13.5)	143.0 (12.7)	37.0 (13.7)
**Polypharmacy**			
No	236 (72.6)*	693 (61.9)	130 (48.0)
Yes	89 (27.4)	427 (38.1)	141 (52.0)*
**Health problems**			
None	50.0 (15.4)*	91.0 (8.1)	21.0 (7.7)
Hypertension	185.0 (56.9)	690.0 (61.6)	190.0 (70.1)*
Diabetes	71.0 (21.8)	321.0 (28.7)	92.0 (33.9)*
Osteoarthritis	88.0 (27.1)	510.0 (45.5)*	125.0 (46.1)
Osteoporosis	39.0 (12.0)	163.0 (14.6)	50.0 (18.5)
Depression	10.0 (3.1)	113.0 (10.1)	36.0 (13.3)*
Dementia	7.0 (2.2)	11.0 (1.0)	13.0 (4.8)*
Parkinsonism	0 (0)	8 (0.7)	6 (2.2)*
Other diseases	68.0 (20.9)	273.0 (24.4)	78.0 (28.8)

a Compared to non-frail and prefrail;

b Compared to non-frail and frail;

c Compared to prefrail and frail;

* *p*<0.05, based on adjusted residuals of the chi-square test.

SD, standard deviation; CC, calf circumference; BMI, body mass index; ADL, activities of daily living; IADL, instrumental activities of daily living.

**Table 4:** Multinomial logistic regression (odds ratio and 95% confidence intervals) of the association between frailty status and sociodemographic characteristics, clinical aspects, and functional capacity and functionality (n=1,716).

Variables	Non-frail	Prefrail	Frail
	OR (95% CI)	OR (95% CI)	OR (95% CI)
**Sociodemographic and clinical aspects**			
Age (years)	1.00	1.04 (1.01-1.06)**	1.04 (1.01-1.07)**
Health problems (n)	1.00	1.14 (1.02-1.26)*	1.21 (1.06-1.40)**
BMI (kg/m^2^)	1.00	1.17 (1.12-1.23)**	1.28 (1.20-1.35)**
CC (cm)	1.00	0.89 (0.85-0.94)**	0.82 (0.76-0.87)**
**Functional capacity**			
ADL (score)	1.00	0.74 (0.57-0.96)*	0.68 (0.49-0.92)*
IADL (score)	1.00	1.01 (0.95-1.07)	0.94 (0.87-1.02)
Lower extremity strength performance (seconds)	1.00	1.09 (1.04-1.15)**	1.19 (1.12-1.26)**
Functional mobility (seconds)	1.00	1.07 (0.99-1.15)	1.15 (1.06-1.25)**

OR, odds ratio; 95% CI, 95% confidence interval; BMI, body mass index; CC, calf circumference; ADL, activities of daily living; IADL, instrumental activities of daily living;

* *p*<0.05;

** *p*<0.01.
